# MiR-152-5p suppresses osteogenic differentiation of mandible mesenchymal stem cells by regulating ATG14-mediated autophagy

**DOI:** 10.1186/s13287-022-03018-4

**Published:** 2022-07-26

**Authors:** Shaoming Li, Ling Gao, Weidong Zhang, Yanbin Yu, Jingjing Zheng, Xiao Liang, Shanshan Xin, Wenhao Ren, Keqian Zhi

**Affiliations:** 1 Department of Oral and Maxillofacial Surgery, The Affiliated Hospital of Qingdao University, 1677 Wutaishan Road, Huangdao district, Qingdao, 266555 Shandong China; 2 School of Stomatology, Qingdao University, Qingdao, 266003 China; 3 Key Lab of Oral Clinical Medicine, The Affiliated Hospital of Qingdao University, Qingdao, 266555 China; 4 College of Safety and Environmental Engineering, Shandong University of Science and Technology, Qingdao, 266590 China; 5 Department of Endodontics, The Affiliated Hospital of Qingdao University, Qingdao, 266003 China; 6 Department of Neurology, Haukeland University Hospital, 5021 Bergen, Norway

**Keywords:** miR-152-5p, ATG14, ROS, Autophagy, Osteoporosis

## Abstract

**Background:**

Osteoporosis affects the mandible resulting in bone loss. Though impairments are not life threatening, they affect a person's quality-of-life particularly vulnerable elderly. MicroRNAs (miRNAs) are novel regulatory factors that play an important role in regulating bone metabolism. Autophagy is evolutionarily conserved intracellular self-degradation process and is vital in the maintenance of both miRNA and bone homeostasis. However, the role of autophagy in the pathogenesis of miRNA regulating osteoporosis remains unclear.

**Methods:**

In the study, we established a rat osteoporosis model induced by ovariectomy (OVX) and isolated mesenchymal stem cells from mandible (MMSCs-M). Several miRNAs were identified to regulate osteoporosis in some studies. qRT-PCR was applied to examine the expression of miRNA, autophagy and osteogenic differentiation-related genes. Western blotting assays were performed to detect the expression of autophagy and osteogenic differentiation proteins. Immunofluorescence and transmission electron microscope were used to verify the autophagy activity. Transfecting technology was used to enhance or suppress the expression of miR-152-5p which enable us to observe the relationship between miR-152-5p, autophagy and osteogenic differentiation. Additionally, the measurement of reactive oxygen species was used to investigate the mechanism of autophagy affecting osteogenic differentiation.

**Results:**

We found an upregulated expression of miR-152-5p in MMSCs-M in OVX group. Downregulated autophagy-related gene, proteins and autophagosome were detected in *vitro* of OVX group compared with sham group. Moreover, downregulation of miR-152-5p promoted osteogenic differentiation of MMSCs-M as well as enhanced autophagy-related proteins in OVX group. Conversely, overexpression of miR-152-5p showed opposite effect in sham group. Meanwhile, we found *Atg14* (autophagy-related protein homolog 14) was identified to be a direct target of miR-152-5p theoretically and functionally. In other words, we confirmed inhibition of miR-152-5p promoted the osteogenic differentiation via promoting ATG14-mediated autophagy. Furthermore, miR-152-5p/ATG14-mediated autophagy regulated osteogenic differentiation by reducing the endogenous ROS accumulation and maintaining cellular redox homeostasis.

**Conclusion:**

Our data suggest that miR-152-5p is the first identified to regulate osteogenic differentiation by directly targeting autophagy-related protein ATG14 and regulating oxidative stress and therapeutic inhibition of miR-152-5p may be an efficient anabolic strategy for osteoporosis.

**Supplementary Information:**

The online version contains supplementary material available at 10.1186/s13287-022-03018-4.

## Introduction

Osteoporosis (OP) is a systemic skeletal disease characterized by low bone mass and microarchitectural deterioration of bone tissue with a consequent increase in bone fragility and susceptibility to fracture. Studies have found that bone loss in the mandible from osteoporosis is linked to several oral diseases, like periodontal diseases and dentition defect [1]. Besides, OP impacts on periodontal and implant therapy due to that the rate of bone-to-implant contact, bone support and implant survival may be impaired in patients with osteoporosis [2]. However, there is currently no cure for osteoporosis.

Bone marrow mesenchymal stem cells (BMMSCs) is a type of progenitors of osteoblasts and adipocytes [3]. BMMSCs possess the potential of multi-directional differentiation and hold prominent immunosuppressive capability, which facilitates clinical application of BMMSCs in immune diseases. Growing evidence has shown the impaired function of BMMSCs is a determinant in the development of OP [4, 5]. Although BMMSCs have been extensively explored in bone tissue engineering, mesenchymal stem cells from mandible (MMSCs-M) have been reported to possess higher potency for osteoblastic differentiation and linked to osteoporosis in the mandible [6].

Autophagy is a physiological cellular process for the degradation and elimination of misfolded proteins and damaged organelles that functions in response to some stimulus, such as stress [7], starvation [8], cell death [9] and tumor suppression [10]. Autophagy has a vital role in physiological conditions and is associated with certain diseases, including cancer and diabetes. Increasing evidence in recent years has implicated autophagy is involved in the development of OP [11, 12]. Autophagy protects from osteoporosis by inhibiting the osteoclast genesis [13]. Besides, it also enhances the survival and function of osteoblasts [14]. Deactivation of autophagy in osteoblasts can decrease mineralizing capacity and induce imbalance in bone formation by osteoblasts and bone resorption by osteoclasts, leading to a loss of bone mass [15].

MicroRNA(miRNA) is a small endogenous non-coding RNA that plays a critical role in both physiological and pathological processes, including cell proliferation [16], apoptosis [17] and differentiation [18]. In addition, miRNA plays an essential role in the regulation of bone formation and homeostasis. Several miRNAs were shown to regulate osteoporosis, such as miR-451a [19], miR-376c [20], miR-765 [21]. MiRNAs regulate elements of the autophagic process, autophagy itself can also mediate miRNAs and their production.

In this study, we constructed mandibular osteoporotic rat model by bilateral ovariectomy and selected ten miRNAs that were reported to regulate autophagy, including miR-20a-5p [22], miR-125a-5p [23], miR-146a-5p [24], miR-378-3p [25], miR-152-5p [26], miR-100-5p [27], miR-140-3p [28], miR-542-3p [29], miR-96-5p [30], miR-185-5p [31]. We revealed an upregulated miR-152-5p level in maxilla, mandible and MMSCs-M of ovariectomized rats compared with sham group. MiR-152 is abnormally expressed in a variety of diseases, including various cancers. Previous study revealed the role of miR-152-5p as a microRNA passenger strand and a tumor suppressor in human gastric cancer cells [32]. Nonetheless, the functions of miR-152-5p in OP and its linkage with autophagy remain underexplored. Hence, we further demonstrated that downregulation of miR-152-5p stimulates osteogenic differentiation of OVX MMSCs-M by promoting ATG14-mediated autophagy which could maintain cellular redox homeostasis. Our findings suggest that ATG14-mediated autophagy regulates the regenerative function of MMSCs-M and controls the development of OP. Our data suggest that miR-152-5p is the first identified to regulate osteogenic differentiation by directly targeting autophagy-related protein ATG14 and regulating oxidative stress, and inhibition of miR-152-5p may provide an effective therapeutic method for osteoporosis.

## Materials and methods

### Animals

All female Sprague–Dawley rats were purchased from Animal Experimental center of Shandong University. Two-month-old female Sprague–Dawley rats were randomly divided into two groups. One group was received by bilateral OVX surgery, another five rats were performed by sham operation, the same treatment without surgical removal of the ovaries. All rats were under standard feeding condition.

### Micro-computed tomography and bone histomorphometry

Micro-computed tomography images were implemented on Mandible to analyze bone mass using Micro-computed tomography. The parameters were set at 80 kv, 500-uA microfocus. The slices images were reconstructed by AVZIO software to produce the three-dimensional images. The CT value was used to estimate the BMD by establishing the standard body model. Bone volume relative to total volume (BV/TV) and trabecular thickness (Tb. Th.) were analyzed by ImageJ software through establishing the region of interest (ROI). Mandible tissue sections were stained by the hematoxylin and eosin (H&E) to test the morphology of bone. The results were observed by the microscope.

### Isolation, culture, purification and characterization of MMSCs-M

Two months after the sham and OVX surgical operation, sham and OVX MMSCs-M were isolated from the mandibles of rats and cultured. In brief, the soft tissues, the whole teeth and the periodontal tissue were removed. Then, the mandibles were broken with scissors and washed the marrow cavities with MEM (HyClone, USA), added with 10% FBS (HyClone, USA) and 1% penicillin and streptomycin (Solarbio, China). The cell suspension was purified by gradient centrifugation and seeded into culture flask. The medium was changed every three days. After 7–10 days, the adherent and confluent cells were digested with trypsin to passage one. MMSCs-M which at passage 1–3 were used for the tests.

A flow cytometer (Becton–Dickinson) was used to test phenotype of MMSCs-M. CD45, CD90 and CD11b (FITC-conjugated) (Elabscience, China) monoclonal antibodies were used to dye cells for 30 min at 4 °C. FlowJo_v10 was applied to process the data.

### Transfection and osteogenic induction of MMSCs-M in vitro

To detect the role of miR-152-5p and ATG14 in osteogenic differentiation of MMSCs-M, miR-152-5p mimics, inhibitors, siATG14 and corresponding negative controls (NC) were designed and accomplished by GenePharma, China. The sequences of miR-152-5p mimics and inhibitors are presented in Table 1. When the cells grew to 60%, mimics, inhibitors or NC were transfected into MMSCs-M, respectively, by using lip3000 (Invitrogen, USA). The sequence is presented in Table 1. After transfecting 48 h, normal culture medium (MEM, added with 10% FBS and 1% penicillin and streptomycin) was replaced by osteogenic induction medium (10%FBS, 1% penicillin–streptomycin, MEM, 100 mmol/L dexamethasone, 0.05 mmol/L ascorbic acid, 10 mmol/Lβ-glycerophosphate). The cells were cultured for 14 days and the osteogenic induction medium was changed every 3 days.

**Table 1:** The sequence of primers

Reagents	Sequence 5'to 3'
Negative control	Sense UUCUCCGAACGUGUCACGUTT
	Antisense ACGUGACACGUUCGGAGAATT
Inhibitor NC	Sense CAGUACUUUUGUGUAGUACAA
miR-152-5p inhibitor	Sense AGUCGGAGUGUAUCACAGAACCU
miR-152-5p mimics	Sense AGGUUCUGUGAUACACUCCGACU
	Antisense UCGGAGUGUAUCACAGAACUUUU
Rat-β-actin	Sense CUCUGAACCCUAAGGCCAATT
	Antisense UUGGCCUUAGGGUUCAGAGGG
Atg14-rat-250	Sense GGGAGAGGUUUAUUGACAATT
	Antisense UUGUCAAUAAACCUCUCCCTT
Atg14-rat-614	Sense GCGGAGGUUACACAUCCUATT
	Antisense UAGGAUGUGUAACCUCCGCTT
Atg14-rat-1092	Sense GCAAACAUCCUUUACCUUUTT
	Antisense AAAGGUAAAGGAUGUUUGCTT

### ALP activity assay ALP staining and Alizarinred S staining

PBS was used to wash the cells three times after completed the induction program and then cell lysis buffer (Beyotime, P0013J, China) was put to extract the protein sample. ALP activity was measured by using ALP activity (Beyotime, P0321, China). Following the instruction, 1ul protein sample was added to 96-well plates and incubated for 10 min. A standard curve was made to normalize the protein concentration. The samples were measured by microplate reader at 405 nm. Similarly, ALP staining and Alizarin Red S staining were performed by Alkaline Phosphatase kit (Meilunbio, China) and Alizarin Red solution (Solarbio, China). 4% paraformaldehyde was used to fix the cells and staining reagents were co-incubated with the cells for 10 min. Finally, the results were observed by the microscope.

### Adipogenic differentiation and oil red O staining assay

MMSCs-M were cultured with adipogenic induction medium (ScienCell, China) or Chondrogenic differentiation medium (Procell, China) for 14 days. Oil Red O staining assay (VivaCell Bioscience) and Chondro-dye (VisaCell Biosciences) were applied to detect the ability of adipogenic and Chondrogenic differentiation following the instruction. Finally, the pictures were observed by the microscope.

### Immunofluorescence analysis

Cells were put into 24-well plates which have coverslips. When the cells grew 50–60%, some treatments were performed on cells. After 48 h, the medium was removed and PBS was used to wash the coverslips with cells. 4% paraformaldehyde was used to fix the cells and then the cells were permeabilized with 0.1%Triton X-100 for 10 min. After that, the cells were blocked by 5% bovine serum albumin and incubated with LC3 (1:200, Proteintech) antibody at 4℃ overnight. On the second day, the anti-rabbit second antibody was incubated in the cells for 1 h and the cell nucleus was stained by DAPI for 5 min. Images were taken by laser scanning confocal microscope.

### Transmission electron microscope

The pre-treated cells were harvested and fixed in the Glutaraldehyde, 2.5% (Solarbio). The next steps were accomplished by the experts and the images were observed with a transmission electron microscope (JEM-1200, Jeol, Japan).

### Western blotting assay

All cells were washed with PBS three times and then treated with Cell lysis buffer for Western and IP (Beyotime Biotechnology) at the iceberg for 1 h. We use the BCA Protein Assay Kit (solarbio) to determine the protein concentration. Protein samples of equal mass were loaded onto SDS-containing polyacrylamide gels, after electrophoresis, then the protein sample was transferred to the polyvinylidene fluoride (PVDF) membrane (Sigma-Aldrich, China). The membrane was sealed for 2 h with skim milk mixed with PBS‐Tween 20 (PBST) at room temperature. Then, the above primary antibodies were incubated overnight at 4 ℃. The membrane was rinsed with PBST three times, and the corresponding secondary antibody was incubated at room temperature for 1 h. Finally, the image was taken by the ChemiDoc Touch Imaging System (Bio-Rad). Bands were quantified by ImageJ software (National Institutes of Health). The reagents are presented in Table 2.

**Table 2:** Reagents information

Reagents	CAT number	Company
Runx2	EPR22858-106	Abcam
ALP	EPR4477	Abcam
OCN	SC-390877	Santa Cruz Biotechnology
LC3	14,600–1-AP	Proteintech
GAPDH	10,494–1-AP	Proteintech
ATG14	28,021–1-AP	Proteintech

### Real-time PCR

Total RNA was extracted from cells or mandible bone tissues and cells using TRIzol reagent (Life Technologies, USA). cDNA was synthesized using a PrimeScript RT reagent kit (TaKaRa, Dalian, China) following the instructions. The real-time PCR was performed using a CFX96 Real-Time System (Bio-Rad) with SYBR Premix Ex Taq (TaKaRa). GAPDH or U6 was aided as internal control. The consequences of each gene were analyzed by the 2-△△Ct method. The primers are listed in Table 3.

**Table 3:** The sequence of primers

Gene	Sequence
Runx2	Forward: CTTCAAGGTTGTAGCCCTCG
	Reverse: TAGTTCTCATCATTCCCGGC
ALP	Forward: CTAGTTCCTGGGAGATGGTA
	Reverse: GTGTTGTACGTCTTGGAGAGA
OCN	Forward: CATGAGGACCCTCTCTCTGC
	Reverse: TGGACATGAAGGCTTTGTCA
ATG5	Forward: TGGGATTGCAAAATGACAGA
	Reverse: TTCCCCATCTTCAGGATCAA
LC3	Forward: TACCAAGGCAAAAAGGGACG
	Reverse: CCCCTGACACTGCTCTTCTAT
P62	Forward: AGCTGCCCTCAGCCCTCT
	Reverse: GGCTTCTCTTCCCTCC
GAPDH	Forward: CCTCGTCTCATAGACAAGATGGT
	Reverse: GGGTAGAGTCATACTGGAACATG
ATG14	Forward: TGCCGAACAATGGGGACTAC
	Reverse: AGGCAGGGTTGTTATGCTCC
miR-152-5P	Forward: GCCATTAGGTTCTGTGATACACTCC
	RT: GTCGTATCCAGTGCAGGGTCCGAGGTATTCGCACTGGATACGACagtcgg
miR-20a-5p	Forward: GGGCCTAAAGTGCTTATAGTGCAG
	RT: GTCGTATCCAGTGCAGGGTCCGAGGTATTCGCACTGGATACGACctacct
miR-125a-5p	Forward: GGTCCCTGAGACCCTTTAACCT
	RT: GTCGTATCCAGTGCAGGGTCCGAGGTATTCGCACTGGATACGACtcacag
miR-146a-5p	Forward: GGGCTGAGAACTGAATTCCATG
	RT: GTCGTATCCAGTGCAGGGTCCGAGGTATTCGCACTGGATACGACaaccca
miR-378-3p	Forward: GGGCACTGGACTTGGAGTCAG
	RT: GTCGTATCCAGTGCAGGGTCCGAGGTATTCGCACTGGATACGACccttct
miR-100-5p	Forward: GGGAACCCGTAGATCCGAACT
	RT: GTCGTATCCAGTGCAGGGTCCGAGGTATTCGCACTGGATACGACcacaag
miR-140-3p	Forward: GGGCTACCACAGGGTAGAACC
	RT: GTCGTATCCAGTGCAGGGTCCGAGGTATTCGCACTGGATACGACccgtgg
miR-542-3p	Forward: GGGCCTGTGACAGATTGATAACT
	RT: GTCGTATCCAGTGCAGGGTCCGAGGTATTCGCACTGGATACGACtttcag
miR-96-5P	Forward: GGGTTTGGCACTAGCACATTTT
	RT: GTCGTATCCAGTGCAGGGTCCGAGGTATTCGCACTGGATACGACagcaaa
miR-185-5p	Forward: GCGTGGAGAGAAAGGCAGTT
	Reverse: GTCGTATCCAGTGCAGGGTCCGAGGTATTCGCACTGGATACGACtcagga
U6	Forward: CGCTTCGGCAGCACATATACTA
	RT: GGAACGCTTCACGAATTTGC
Universe R	CCAGTGCAGGGTCCGAGGT

### Luciferase reporter assay

ATG14, the candidate targets of miR-152-5p, was predicted by TargetScan and miRBD. The binding sequence between them was predicted by TargetScan and miRDB. To detect whether miR-152-5p could bind the 3′UTR of Atg14 mRNA, we performed the luciferase reporter assay. The WT (wild-type) 3’-UTR or MUT (mutant) 3’-UTR of Atg14 was designed and cloned to EGFP plasmids (Genechem, Shanghai, China). Then, miR-152-5p plasmids or NC along with WT or MUT 3’-UTR plasmids were co-transfected into MMSCs-M. After 48 h of transfection, dual-Luciferase Reporter Assay System (Promega, USA) was used to analyze the luciferase activity.

### Measurement of ROS

The qualitative and quantitative ROS levels on MMSCs-M were measured by different assays, respectively. MMSCs-M were seeded in 96-well plates and cultured for 24 h. After reached 70%, the cells were transfected as described before and then cultured for 48 h. An ROS test kit (Beyotime, S0033, China) was used to test intracellular ROS level. Following the instruction of the kit, DCFH-DA was added and cultured. After 0.5 h, the reagents were replaced by PBS. Fluorescence microscope and fluorescence microplate were used to test the level of ROS qualitatively and quantitatively.

### Statistical analysis

All data were displayed as mean ± SD. Independent t test and one-way analysis were performed to analyze those data by Graphpad Prism 7.0. If the result is significant analyzed by one-way ANOVA, Bonferroni test is required for a post-test. *p* < 0.05 was considered statistically significant.

## Results

### Inhibition of autophagy and upregulation of miR-152-5p in OVX MMSCs-M

**Fig. 1:**
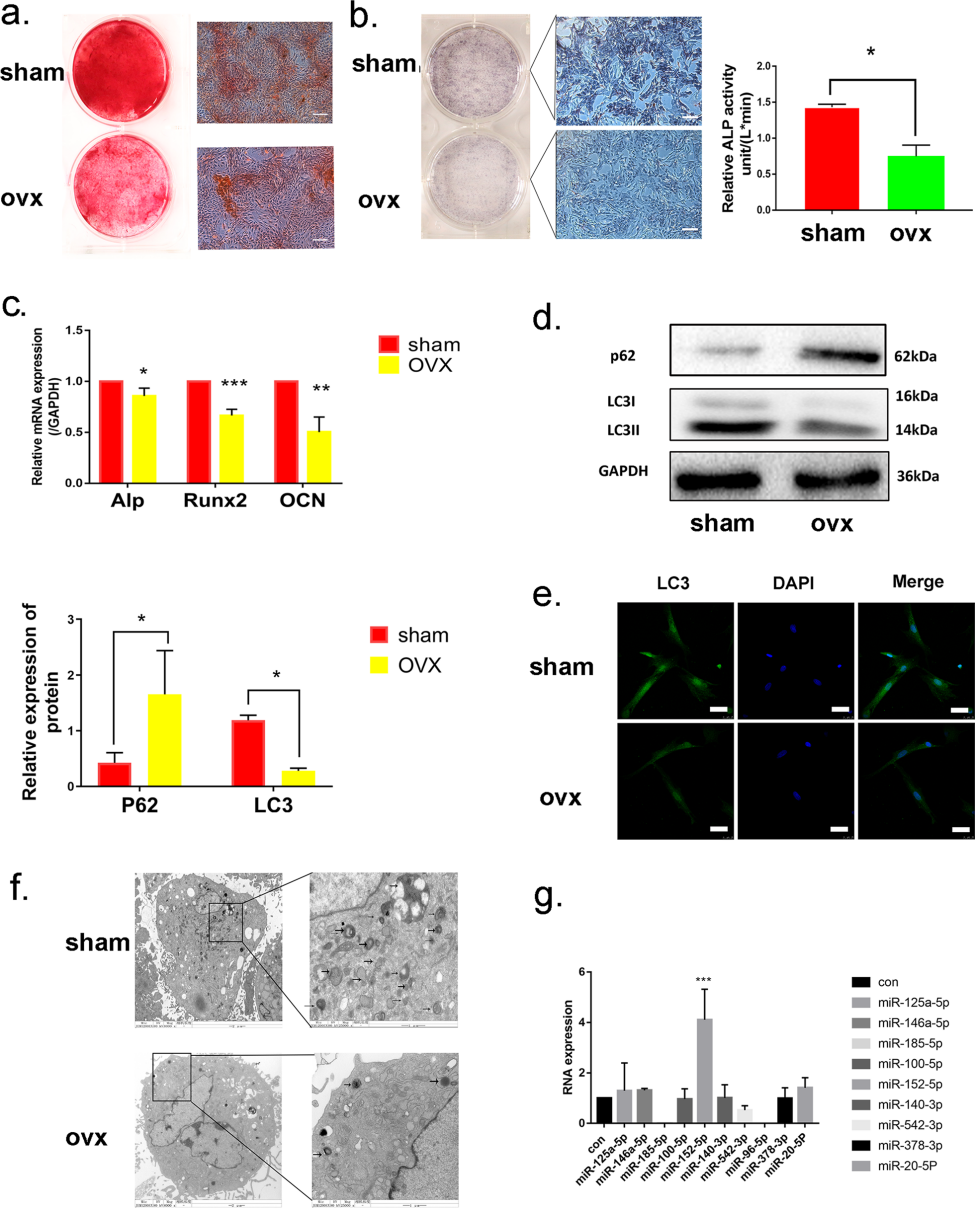
Inhibition of autophagy and upregulation of miR-152-5p in OVX MMSCs-M. **a** Alizarin Red S staining was performed to illustrate mineralized nodule after 14 days of osteogenic induction, scar bar = 200um. **b**. ALP staining and ALP activity assays were performed on 7 days of osteogenic induction, scar bar = 200um. **c** QRT-PCR and western blots assay were performed to detect the osteogenesis-related markers including *Runx2*, *Alp* and *Ocn* (n = 3) after 14 days of osteogenic induction at RNA and protein level. **d** The expression of LC3B and p62 protein (n = 3) in OVX and sham MMSCs-M were detected by western blots assays after 14 days of osteogenic induction. **e** Immunofluorescence detected the protein LC3B (green) in sham and OVX MMSCs-M with blue (DAPI staining) nucleus. Scar bar = 50um. **f** The number of autophagic vacuoles was observed by TEM, scar bar = 2um. **g** The expression of ten miRNAs in OVX and sham MMSCs-M. Data were shown as mean ± SD (*p* < 0.05*, *p* < 0.01**, *p* < 0.0005***, *p* < 0.0001****)

Initially, the mandibular osteoporotic rat model was established by bilateral ovariectomy. Micro-computed tomography analysis and H&E staining revealed that the bone mass of mandible in OVX rats significantly decreased compared with the sham group (Additional file 1: Fig. S1a–b). In order to investigate the mechanism of bone loss in mandibles of OVX rats, MMSCs-M were then isolated from the mandibles of OVX and sham rats and maintained in vitro culture system. Cell identities were examined via flow cytometric analysis, MMSCs-M from both OVX and sham rats were positive for CD90, but negative for CD45, CD11b (Additional file 1: Fig. S1c). We detected the capacity of osteogenic differentiation of sham and OVX MMSCs-M through Alizarin Red S staining and ALP staining and ALP activity assay. OVX MMSCs-M formed less mineralization nodules than sham group (Fig. 1a), coinciding with the decreased expression and activity of ALP (a marker of osteogenic differentiation at early stage) via ALP staining and ALP activity assay (Fig. 1b). In addition, Oil Red O staining (ORO) and Toluidine Blue O were applied to assess the ability of adipogenic and chondrogenic differentiation. However, there was no significant difference in adipogenic and chondrogenic differentiation between sham and OVX groups (Additional file 1: Fig. S1d–e). Hence, we paid more attention to osteogenic differentiation and detected the expression of osteogenic differentiation and formation markers including Alkaline phosphatase (ALP), Runt-related transcription factor 2 (RUNX2) and Osteocalcin (OCN) at gene levels using qRT-PCR (Fig. 1c). Compared with the sham group, we found that the expression of ALP, RUNX2 and OCN were reduced in OVX MMSCs after 14 days of osteogenic induction compared with sham group. These results showed that the anomalous osteogenic differentiation commitment of OVX MMSCs-M may be involved in the pathogenesis of ovariectomy-induced osteoporosis.

Recently evidence showed that autophagy activity has association with osteogenic differentiation [34]. To investigate the involvement of autophagy in the ovariectomy-induced osteoporosis, we detected the protein LC3B and P62 in sham and OVX' MMSCs-M. The expression of LC3B was decreased to 0.3-fold and P62 increased threefold in MMSCs-M of OVX compared with sham group (Fig. 1d). The immunofluorescent staining showed that the LC3-positive plots were decreased in OVX' MMSCs-M compared with sham' (Fig. 1e). Additionally, analysis of autophagic vacuoles by transmission electron microscopy (TEM) showed loss of autophagosomes in OVX group (Fig. 1f). Taken together, these data revealed that autophagy displays a decreased tendency in OVX MMSCs-M. Which might contribute to the anomalous differentiation commitment.

MicroRNAs are essential for autophagy regulation in many diseases [35], 36. We then asked whether miRNA-mediated autophagy played an important role in the development of osteoporosis. To address this question, we selected 10 miRNAs which were associated with autophagy, including miR-20a-5p [22],miR-125a-5p [23], miR-146a-5p [24], miR-378-3p [25], miR-152-5p [26], miR-100-5p [27], miR-140-3p [28], miR-542-3p [29], miR-96-5p [30], miR-185-5p [31]. QRT-PCR analysis was performed to investigate the differences of these ten miRNAs in MMSC-M of OVX and sham. The results showed miR-152-5p upregulated in OVX group compared to sham group and the expression of 7 miRNAs was unchanged. In addition, miR-96-5p and miR-185-5p were completely lost in expression (Fig. 1g).

Taken together, these findings suggested a negative correlation between miR-152-5p and the autophagy activity and the capacity of osteogenic differentiation in sham and OVX MMSCs-M. Further investigation to the relationship between miR-152-5p and autophagy activity and osteogenic differentiation is clearly warranted.

### Autophagy is required to maintain the osteogenic differentiation of MMSCs-M

**Fig. 2:**
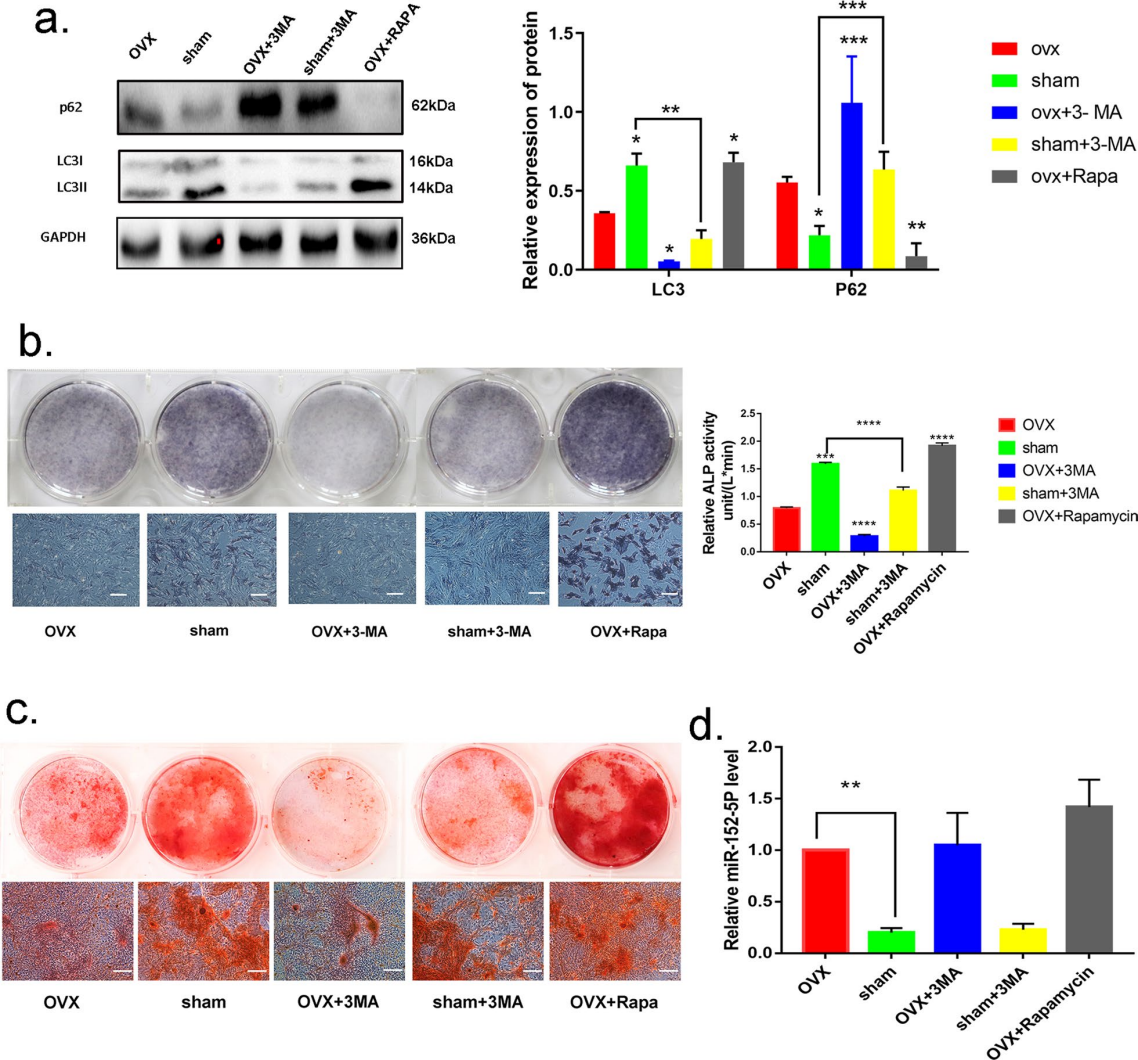
Autophagy is required to maintain the osteogenic differentiation of MMSCs-M. **a** The sham and OVX’ MMSCs-M were treated with 3MA (500uM) and the OVX MMSCs-M were treated with Rapamycin (100 nM) as positive control for 48 h. The expression of LC3B and p62 was assessed by Western blotting assays (n = 3). **b**, **c**. Alizarin Red S staining, ALP staining and activity of these groups. **d** qRT-PCR were performed to detect the expression of miR-152-5p in these groups. scar bar = 200um. Data were shown as mean ± SD (*p* < 0.05*, *p* < 0.01**, *p* < 0.0005***, *p* < 0.0001****)

In order to confirm the linkage between autophagy and osteogenic differentiation, we applied a well-known autophagy inhibitor, 3-methyladenine (3-MA) and an autophagy inducer rapamycin to alter the autophagy activity. Western blotting assessment demonstrated that 3-MA could attenuate the autophagy activity in both OVX and sham MMSCs-M (Fig. 2a), while rapamycin could enhance autophagy (Fig. 2a) which was in line with the previous studies [37]. After 14 days' osteogenic induction, the calcium deposits stained with Alizarin S Red were increased in OVX + rapamycin group compared with OVX group, while 3-MA decreased the number of calcium deposits in sham + 3-MA and OVX + 3-MA groups (Fig. 2c). Meanwhile, the results of ALP staining and activity is in line with variation of calcium deposits in different groups (Fig. 2b). In addition, we also explored whether autophagy affects the expression of miR-152-5p. Neither 3-MA nor rapamycin affected the expression of miR-152-5p, suggesting that autophagy didn't have an influence on the expression of miR-152-5p (Fig. 2d). In summary, these findings indicated that autophagy activity promotes the osteogenic differentiation of MMSCs-M and impaired autophagy has an inhibitory effect on osteogenic differentiation.

### MiR-152-5p inhibits autophagy and osteogenic differentiation in MMSCs-M

**Fig. 3:**
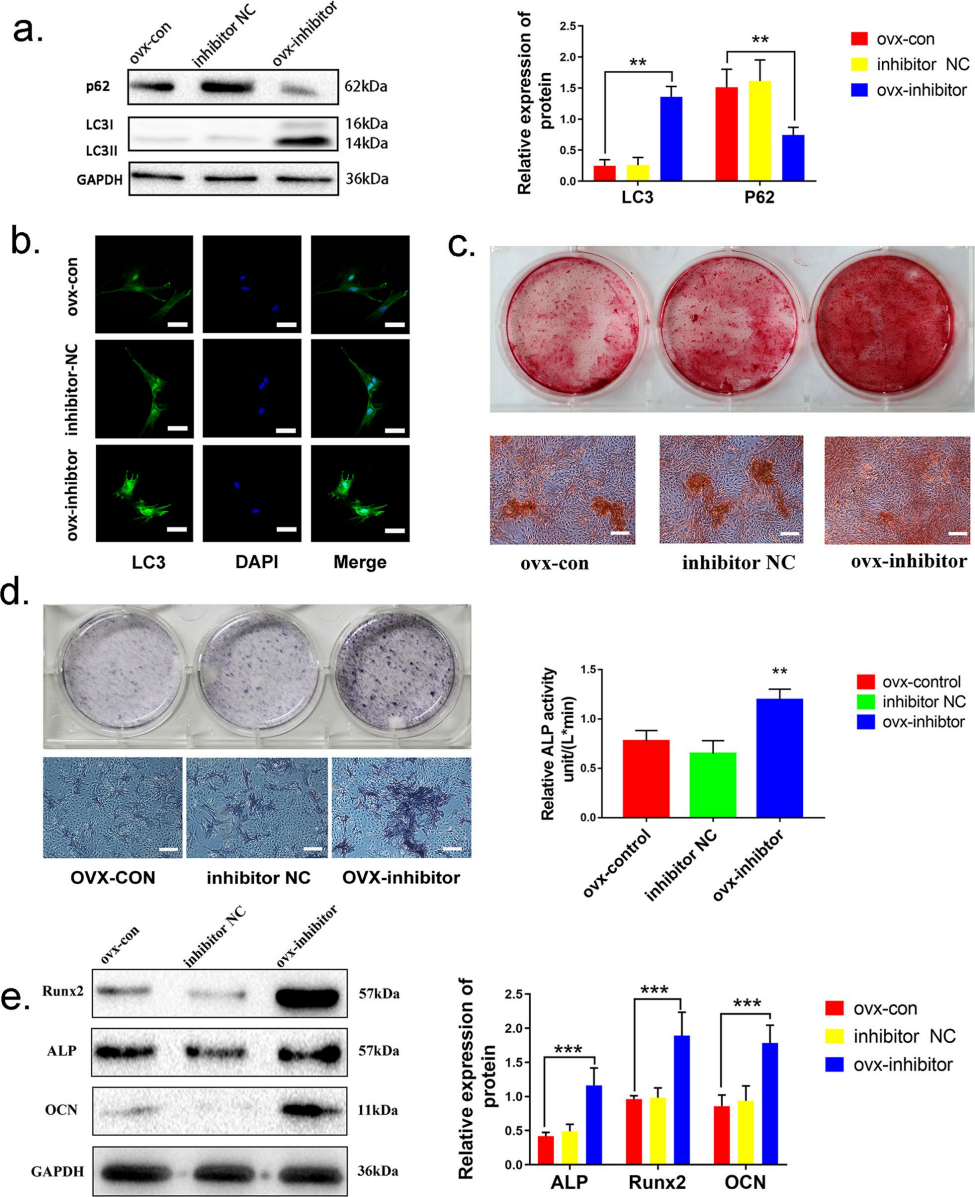
miR-152-5p inhibits autophagy and osteogenic differentiation in MMSCs-M. **a** MiR-152-5p inhibitor and inhibitor NC were transfected into OVX MMSCs-M. Western blots assays detected the level of LC3B and p62 in control, inhibitor, inhibitor NC groups (n = 3). **b** Immunofluorescence staining of LC3B. **c** Alizarin Red S staining was performed to illustrate mineralized nodule in control, inhibitor, inhibitor NC groups. **d** ALP staining and activity of the above groups. **e** The expression of ALP, RUNX2, OCN were detected by western blots assay of these three groups (n = 3). Data were shown as mean ± SD (*p* < 0.05*, *p* < 0.01**, *p* < 0.0005***, *p* < 0.0001****)

To investigate the biological role of miR-152-5p in autophagy activity and osteogenic differentiation in MMSCs-M, miR-152-5p inhibitor was employed to decrease the expression of miR-152-5p in OVX MMSCs-M, while microRNA inhibitor NC (a non-functional gene sequence) and blank control group were used as parallel controls. The suppression efficiency of miR-152-5p inhibitor was measured by qRT-PCR (Additional file 2: Fig. S2a). After transfecting for 48 h, the autophagy-associated markers (LC3B, P62) were measured as described above and the data revealed that downregulation of miR-152-5p increased the autophagy activity (enhanced LC3B and suppressed P62) in OVX MMSCs-M after transfection (Fig. 3a, b). To further validate the result, sham MMSCs-M were transfected with miR-152-5p mimics to upregulate miR-152-5p level. QRT-PCR analysis was used to examine the efficiency of miR-152-5p mimics and showed that the level of miR-152-5p increased in miR-152-5p mimics group compared with NC group (Additional file 2: Fig. S2b–c). However, overexpression of miR-152-5p attenuated the expression of LC3B and increased P62, suggesting upregulated miR-152-5p impaired autophagy activity (Additional file 2: Fig. S2a–b). Collectively, miR-152-5p regulated autophagy activity in MMSCs-M.

In addition, we detected the capacity of osteogenic differentiation in these two groups. The cells were transfected for 48 h, followed by osteogenesis induced for 14 days. Assessment of Alizarin S Red staining revealed that the calcium deposits in miR-152-5p inhibited group were significantly increased compared with blank control or inhibitor NC group (Fig. 3c). The data from ALP activity and staining showed the similar results (Fig. 3d). Additionally, the protein of osteogenic markers including RUNX2, ALP, OCN was also upregulated in the group with miR-152-5p inhibitor (Fig. 3e). Adversely, overexpression of miR-152-5p in sham group suppressed the osteogenic differentiation capacity in sham MMSCs-M (Additional file 2: Fig. S2c–e).

Hence, these results confirmed that downregulation of miR-152-5p significantly promotes the autophagy activity and osteogenic differentiation in MMSCs-M.

### Downregulation of miR-152-5p regulates osteogenic differentiation viapromoting autophagy* in OVX MMSCs-M*

**Fig. 4:**
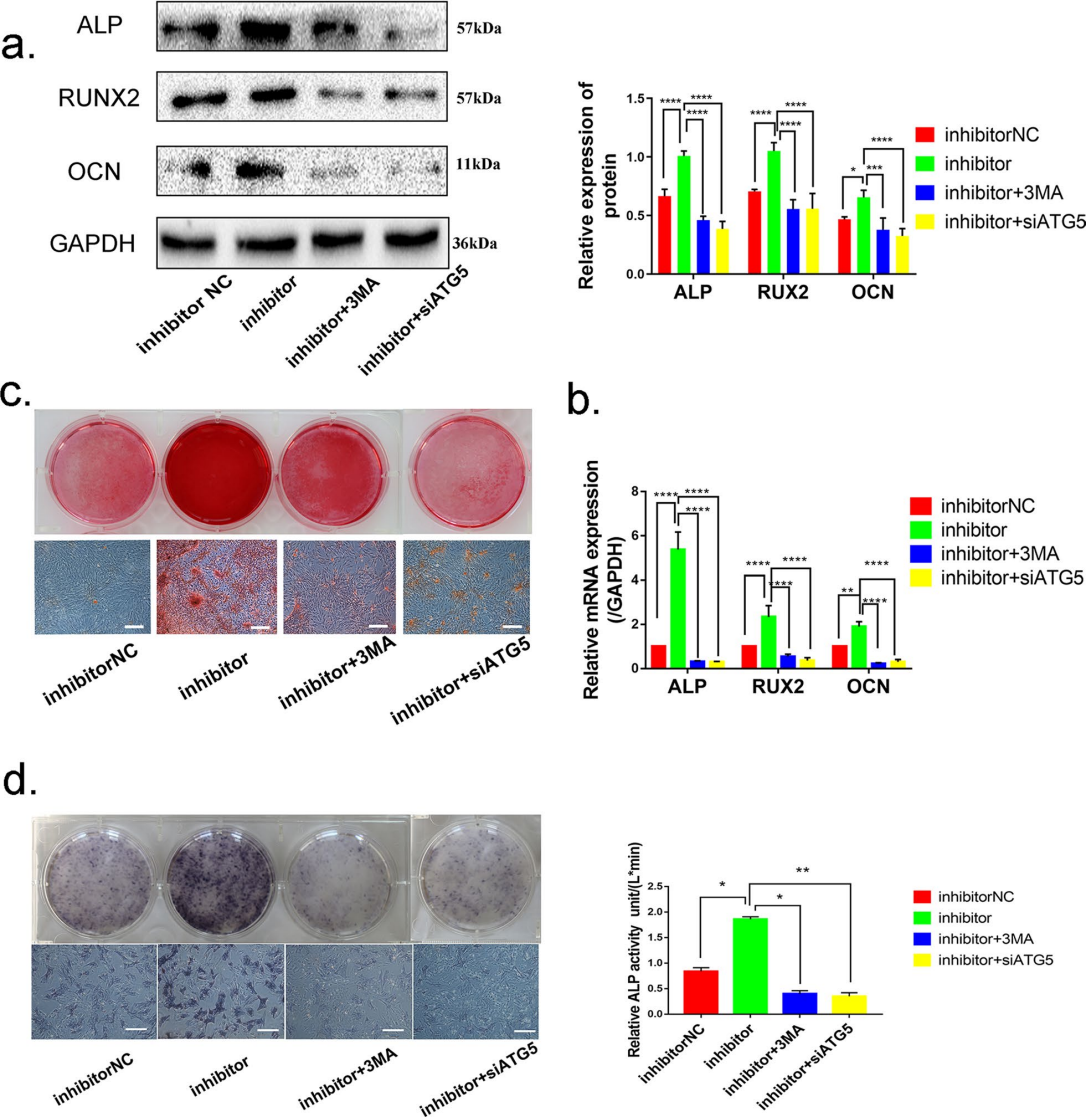
Downregulation of miR-152-5p regulates osteogenic differentiation via promoting autophagy in OVX MMSCs-M. **a** RUNX2, ALP, OCN were detected by Western blots in groups of NC, inhibitor, inhibitor + 3-MA, inhibitor + siATG5 (n = 3). **b** QRT-PCR was performed to assess the expression of *Runx2*, *Alp*, *Ocn* in above groups (n = 3). **c**, **d**. Alizarin Red S staining and ALP staining and activity of these groups. Data were shown as mean ± SD (*p* < 0.05*, *p* < 0.01**, p < 0.0005***, p < 0.0001****)

Based on the results above, we made the hypothesis that miR-152-5p suppressed osteogenic differentiation via regulating autophagy activity in MMSCs-M. Considering 3-MA is an inhibitor of PI3K signal pathway, we wondered whether inhibition of PI3K signal pathway has a direct effect on osteogenic differentiation. In order to minimize this interference, in addition to use 3-MA in OVX MMSCs-M, siAtg5 was applied to downregulate autophagy-related protein homolog (ATG5) after silencing miR-152-5p (Additional file 3: Fig. S3a). Thus, four subgroups of OVX MMSCs-M were further used to explore, including the cells with inhibitor NC, miR-152-5p inhibitor, miR-152-5p inhibitor + 3-MA and miR-152-5p inhibitor + siATG5. The expression of the osteogenic differentiation markers (RUNX2, ALP and OCN) was detected on day 14 of osteoblastic induction. Western blotting and qRT-PCR showed that both 3-MA and siAtg5 abolished the promotion of miR-152-5p inhibitor on osteogenic differentiation, with showing no difference in groups of miR-152-5p inhibitor + 3-MA and miR-152-5p inhibitor + siAtg5 (Fig. 4a, b). Consistently, Alizarin Red S staining and ALP staining showed that the enhanced osteogenic differentiation induced by miR-152-5p downregulation was abrogated by blocking autophagy (Fig. 4c, d). The data revealed that downregulation of miR-152-5p promotes osteogenic differentiation via activating the autophagy.

### *Atg14* is a direct target of miR-152-5p

**Fig. 5:**
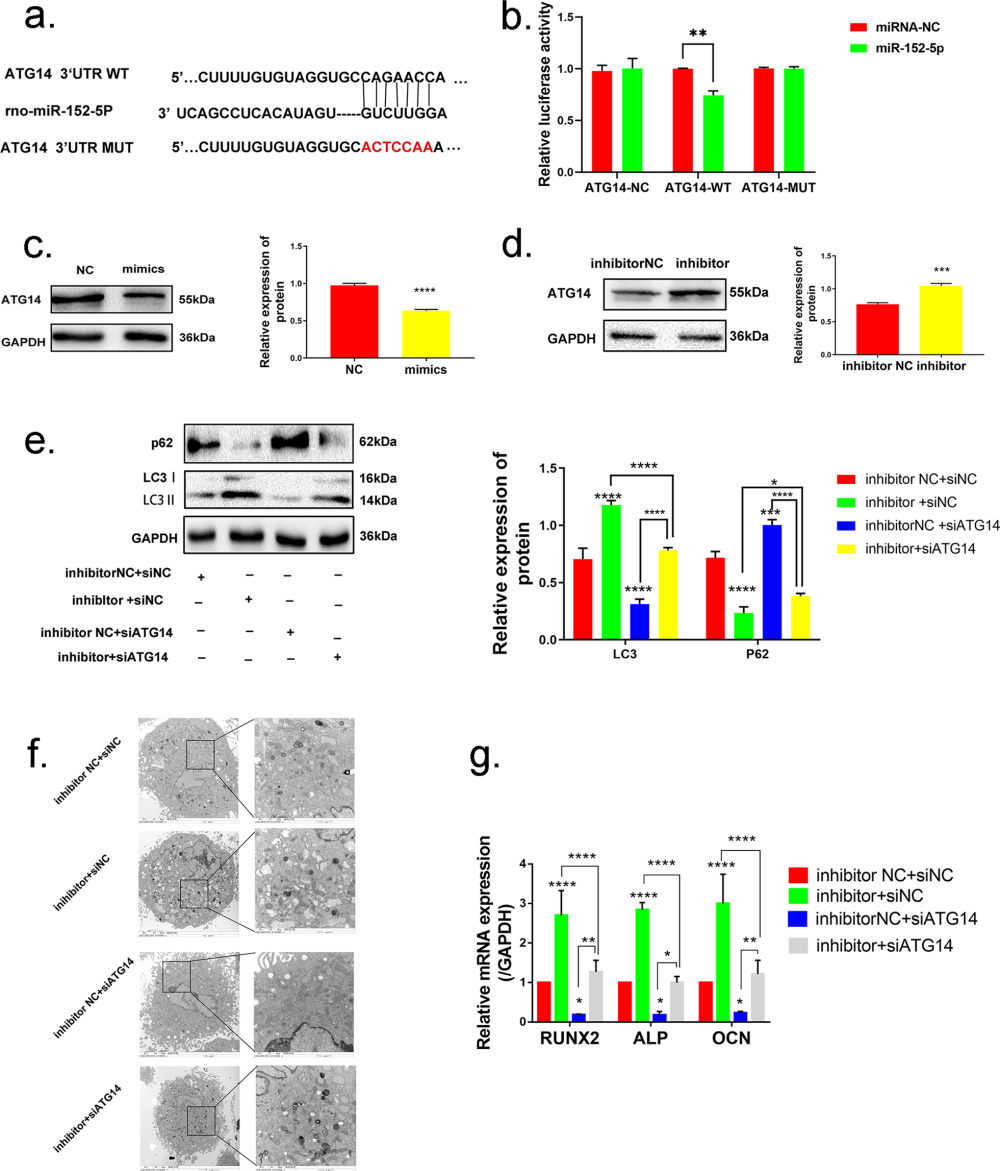
*Atg14* is a direct target of miR-152-5p. **a** The supposed target sequence for miR-152-5p on the 3’-UTR of *Atg14*. **b**, **c** miR-152-5p was overexpressed in sham MMSCs-M by transfecting miR-152-5p mimics, while miR-152-5p inhibitor was used to downregulate the expression of miR-152-5p in OVX MMSCs-M. Western blotting assessed the expression of ATG14 in these groups. **d** Luciferase activity was performed in MMSCs-M. **e** Western blotting assays were performed to the expression of protein LC3B and p62 in the groups of inhibitor NC + siNC, miR-152-5p inhibitor + siNC, inhibitor NC + siATG14, miR-152-5p inhibitor + siATG14 (n = 3). **f** The number of autophagic vacuoles was observed by TEM in the above groups, scar bar = 2um. **g** QRT-PCR was performed to assess the osteogenic markers in the above groups. Data were shown as mean ± SD (p < 0.05*, p < 0.01**, p < 0.0005***, p < 0.0001****)

To illustrate the physiological mechanism underlying miR-152-5p's regulation in autophagy, the candidate targeting genes selected from TargetScan and miRBD (miRNA target prediction databases) were explored. ATG14 was identified as an essential mediator in autophagy [38]. Due to the high-potential binding sites, *Atg14* was considered as the candidate gene for miR-152-5p (Fig. 5a). Luciferase activity was performed to investigate whether miR-152-5p directly binds to the 3′UTR of Atg14 mRNA. We showed that the expression of wild-type *Atg14* was inhibited by transfecting miR-152-5p plasmids but not in mutant type (Fig. 5b). Additionally, MMSCs-M were used to further verify the functional properties of miR-152-5p on the expression of ATG14. Western blotting assessment showed that ATG14 protein level was significantly decreased when miR-152-5p was overexpressed (Fig. 5c). Meanwhile, the miR-152-5p inhibitor significantly increased ATG14 protein level compared to the group with inhibitor NC (Fig. 5d). These results indicated that miR-152-5p targets *Atg14* directly.

To investigate the functional effect on miR-152-5p targeting *Atg14,* we applied siAtg14 in OVX MMSCs-M. QRT-PCR analysis confirmed a suppressed *Atg14* expression in siAtg14-1902 (Additional file 3: Fig. S3b), siAtg14-1902 was selected for the further functional experiment. Measurements of the autophagy activity were carried out in four subgroups: inhibitor NC + siNC, miR-152-5p inhibitor + siNC, inhibitor NC + siATG14 and miR-152-5p inhibitor + siATG14. As demonstrated by analysis of autophagic vacuoles and the other decreased autophagy-associated markers, siAtg14 treated OVX MMSCs-M displayed a reduced autophagy activity (Fig. 5e, f). Meanwhile, inhibition of *Atg14* also attenuated the promotion of miR-152-5p inhibitor on autophagy. Additionally, we tested the capacity of the osteogenic differentiation in above subgroups and found a decreased autophagy by inhibiting *Atg14* suppressed osteogenic differentiation in OVX MMSCs-M (Fig. 5g) (Additional file 3: Fig. S3c). In order to identify the relationship between miR-152-5p and *Atg14,* we overexpressed *ATG14* and miR-152-5p in sham MMSCs-M and found overexpressed ATG14 could rescue the miR-152-5p mimics suppressed autophagy activity and osteogenic differentiation (Additional file 3: Fig. S3d–e).

Taken together, these data suggested *Atg14* is a direct target of miR-152-5p and downregulated miR-152-5p enhances osteogenic differentiation via promoting ATG14-mediated autophagy.

### miR-152-5p/ATG14-mediated autophagy regulates osteogenic differentiation by reducing the endogenous ROS accumulation and maintaining cellar redox homeostasis

**Fig. 6:**
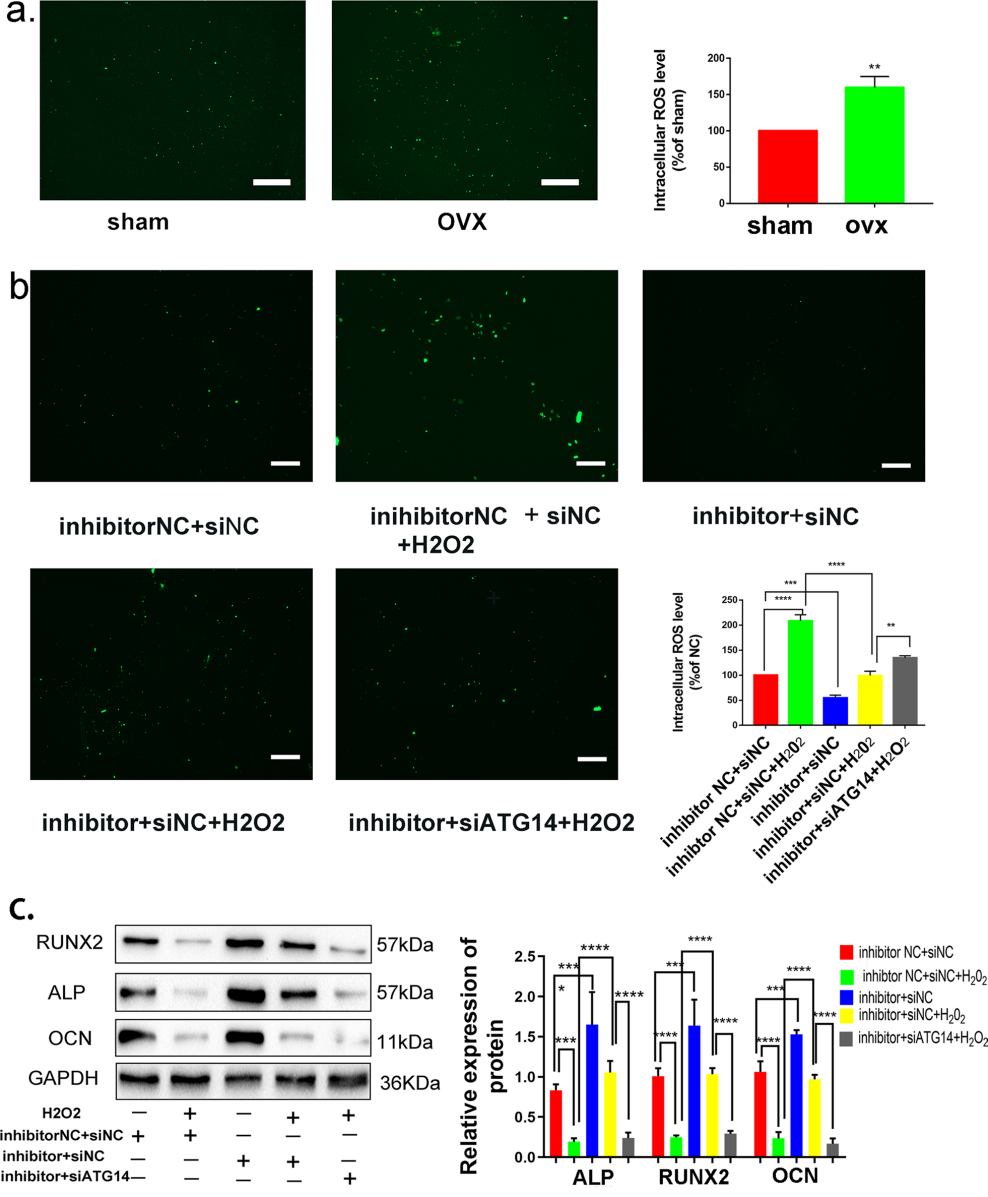
miR-152-5p/ATG14-mediated autophagy regulated osteogenic differentiation by reducing the elevated ROS. **a** ROS detection of sham and OVX’ MMSCs-M. Scar bar = 50um. **b** H_2_O_2_ (100um) was applied to OVX MMSCs-M in order to increase the ROS level. ROS detection of different groups in inhibitor NC + siNC, inhibitor NC + siNC + H_2_O_2_ (100uM), miR-152-5p inhibitor + siNC, miR-152-5p inhibitor + siNC + H_2_O_2_ (100uM), miR-152-5p inhibitor + siATG14 + H_2_O_2_. **c** Western blotting assays were performed to verify that ATG14-mediated autophagy regulated osteogenic differentiation by reducing the elevated ROS (n = 3). Scar bar = 50um. Data were shown as mean SD (*p* < 0.05*, *p* < 0.01**, *p* < 0.0005***, *p* < 0.0001****)

Yet we have found miR-152-5p regulates autophagy by targeting *Atg14*, the possible mechanism of autophagy promoting osteogenic differentiation was unexplored. Accumulating evidence showed that reactive oxygen species (ROS) were involved in the process of osteogenic differentiation [39] [40]. Hence, we investigated the endogenous ROS level in OVX and sham MMSCs-M using DCFH-DA staining. The level of ROS in OVX MMSCs-M was elevated compared with sham group (Fig. 6a). Moreover, to further explore the role of endogenous ROS in miR-152-5p/ATG14-mediated autophagy promoting osteogenic differentiation, H_2_O_2_ (100um) was applied to OVX MMSCs-M in order to increase the ROS level. Then, we checked the ROS level in different subgroups, including inhibitor NC + siNC, inhibitor NC + siNC + H_2_O_2_ (100uM), miR-152-5p inhibitor + siNC, miR-152-5p inhibitor + siNC + H_2_O_2_ (100uM) and miR-152-5p inhibitor + siATG14 + H_2_O_2_. The results showed that treatment with H_2_O_2_ increased the level of endogenous ROS (Fig. 6b). We demonstrated that the level of ROS in miR-152-5p inhibitor + siNC group was lower than that in the inhibitor NC + siNC group (Fig. 6b), with a higher ROS level in the group of inhibitor NC + siNC + H_2_O_2_ than miR-152-5p inhibitor + siNC + H_2_O_2_ group. Meanwhile, compared the ROS level between miR-152-5p inhibitor + siNC + H_2_O_2_ group and miR-152-5p inhibitor + siATG14 + H_2_O_2_ group, we found inhibition of *Atg14* increased the level of ROS, indicating a negative correlation between elevated ROS and autophagy activity. These results suggested that miR-152-5p/ATG14-mediated autophagy can decrease the ROS production in OVX MMSCs-M. To verify that miR-152-5p/ATG14-mediated autophagy regulated osteogenic differentiation by reducing the elevated ROS, we explored the ability of osteogenic differentiation in these groups. The results showed that elevated ROS impaired the ability of osteogenic differentiation and miR-152-5p/ATG14-mediated autophagy attenuated the inhibitory effect of ROS on osteogenic differentiation (Fig. 6c).

**Fig. 7:**
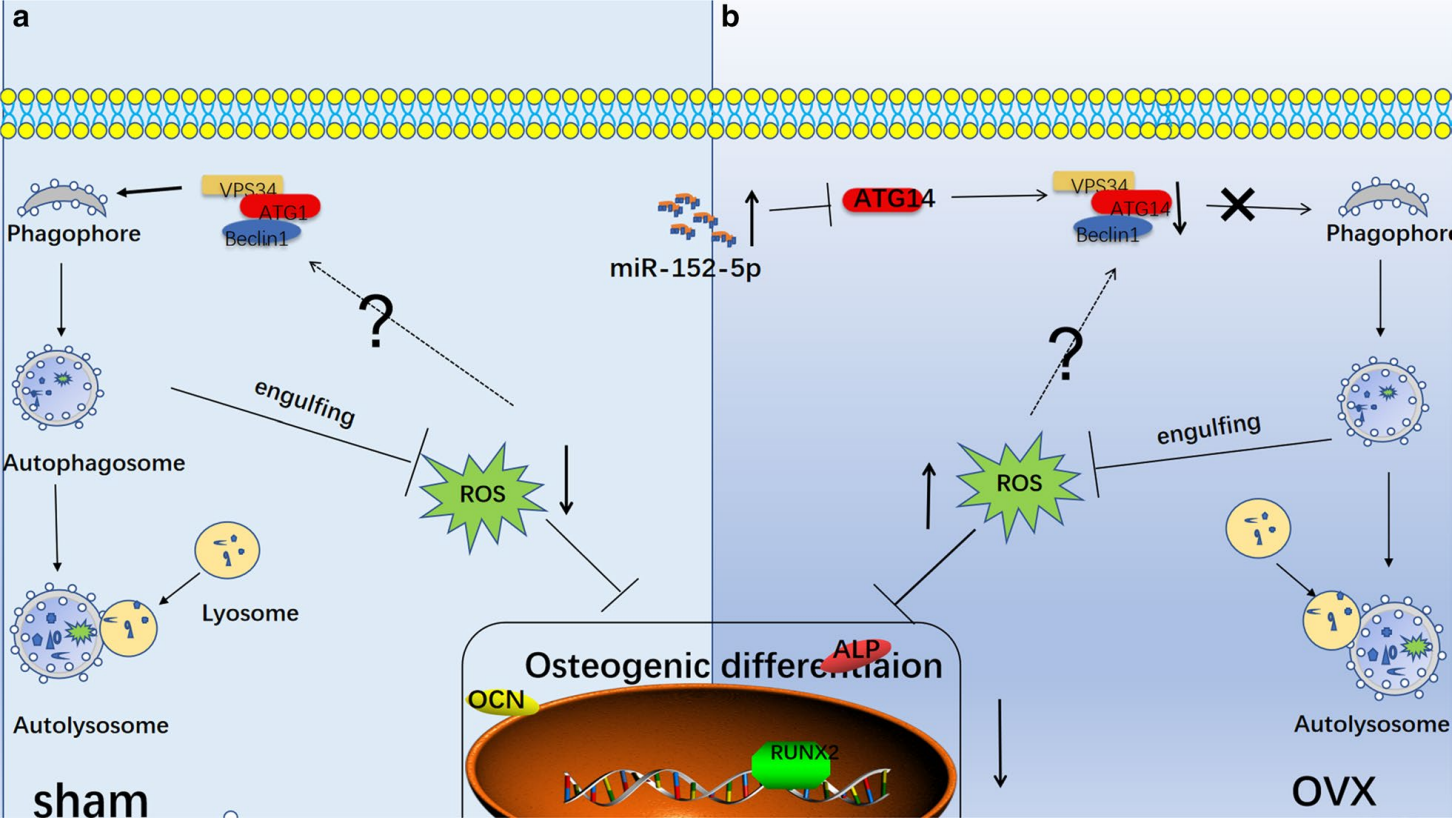
A schematic diagram showing that miR-152-5p suppresses osteogenic differentiation of mandible mesenchymal stem cells by regulating ATG14-mediated autophagy. **a** In sham’ MMSCs-M, autophagy could reduce the elevated ROS and maintain the redox homeostasis. **b** In OVX’ MMSCs-M, the upregulated miR-152-5p has an inhibitory effect on autophagy by targeting *Atg14* and ATG14 interacts with VPS34 and Beclin1 to form ATG14 complex which plays an important role in autophagic initiation, leading to alleviating autophagy activity. The decreased autophagy couldn’t attenuate the inhibitory effect of ROS on osteogenic differentiation

Hence, based on the above data, we concluded that miR-152-5p/ATG14-mediated autophagy can reduce oxidative stress, leading to enhanced osteogenic differentiation (Fig. 7).

## Discussion

OP in the mandible is a bone disease characterized by the mass bone loss and fragility [41]. OP impairs not only the tooth [42] and periodontal tissues [43] but also implantation treatment, especially, causing marginal bone loss around implants [44]. Recently, mounting reports have proved that miRNAs have an effect on osteogenic differentiation of mesenchymal stem cells through transcriptional activation or inhibition of osteogenesis-related genes [45] [46] or the genes in some signal pathway [19]. However, we demonstrated that downregulation of miR-152-5p regulates osteogenic differentiation of OVX MMSCs-M via promoting autophagy. This study provides a new insight for us to explore the pathogenesis of miRNAs regulating osteogenic differentiation and offers a therapeutically target for osteoporosis.

Autophagy is a cell survival pathway and serves a pivotal role in bone homeostasis via degrading and recycling the intracellular components to macromolecular precursors and energy [47]. Autophagy was reported to be as a general house-keeping process which regulates the metabolic status of the cell [48]. A recent report showed that autophagy maintains stem-like features of human gingival mesenchymal stem cells which is instrumental for osteoblastic differentiation [34]. Meanwhile, impaired autophagy induced by HDAC9 (histone deacetylases 9) in mesenchymal stem cells leads to bone loss [49]. In line with those reports, our results illustrated the role of autophagy in the process of osteogenic differentiation and revealed miR-152-5p regulates osteogenic differentiation via suppressing ATG14-mediated autophagy. *Atg14* was identified to be a direct target of miR-152-5p. ATG14 interacts with VPS34 and Beclin1 to form ATG14-containing PtdIns3K complex (phosphatidylinositol 3-kinase) which plays an important role in initiation of autophagosome formation [50]. ULK1 (unc-51 like autophagy activating kinase 1) is a key mediator in the early processes of autophagy induction. ULK1, interacted with ATG13 (Autophagy-related protein homolog 13), enables to phosphorylate ATG14 and activates the ATG14-containing PtdIns3K complex which could promote the initiation of autophagosome formation [50]. Additionally, ATG14 promotes autophagosome–endolysosome fusion via an autophagy-specific membrane fusion mechanism [51]. Those results illustrated that ATG14 plays an important role in the process of autophagy activity which is the reason we selected *Atg14* as the target gene of miR-152-5p. Recent reports showed that ATG14-mediated autophagy is involved in disease pathophysiology such as hepatic insulin sensitivity [52] and neuroinflammation and neuropathic pain [53]. To our knowledge, miR-152-5p/ATG14-mediated autophagy is reported firstly to play a pivotal role in osteogenic differentiation.

ROS, as short-lived oxygen-containing molecules, display high chemical reactivity toward DNA, RNA, proteins and lipids [39]. The relative excessive accumulation of ROS affects cellular homeostasis and serves as prime modulators of cellular dysfunction which contributes to disease pathophysiology [54]. Elevated ROS was defined as oxidative stress and ovariectomy was thought to induce oxidative stress [55]. Hence, we tested the level of ROS in sham and OVX' MMSCs-M and found OVX' MMSCs-M had higher level of ROS than that of sham group. A previous report illustrated that MSCs have lower antioxidant activity and be more susceptible to the injury of oxidative stress compared with differentiated cell types [56]. Additionally, ROS was reported to inhibit the osteogenic differentiation [57]. In our data, we revealed that miR-152-5p/ATG14-mediated autophagy regulated osteogenic differentiation by reducing the endogenous ROS accumulation and maintaining cellular redox homeostasis which was in line with previous reports.

Cellular redox homeostasis plays an important role in maintaining physiological responses and there are multiple pathway to keep the homeostasis [58]. As a catabolic process, autophagy alleviates oxidative damage by engulfing and degrading oxidized substance [59]. Several pathways were reported to take part in this process, such as chaperone-mediated autophagy pathway, mitophagy pathway and P62 delivery pathway [59]. Our results confirmed that miR-152-5p/ATG14-mediated autophagy could maintain cellar redox homeostasis by alleviating oxidative stress, causing to promote osteogenic differentiation in OVX MMSCs-M.

Although we have validated that miR-152-5p/ATG14-mediated autophagy regulates osteogenic differentiation of MMSCs-M via maintaining cellar redox homeostasis, there are still questions which need to be further explored. Increasing studies have reported a crosstalk between autophagy and ROS [60]. As signaling molecules in several pathways, ROS could induce autophagy by several distinct mechanisms [61]. Whether ROS activates miR-152-5p/ATG14-mediated autophagy? Additionally, our present studies were based on the cellular level and animal experiment needs to be considered in further exploration.

## Conclusion

In summary, our results showed an inhibitory role of miR-152-5p in osteogenic differentiation of MMSCs-M by suppressing ATG14-mediated autophagy. Meanwhile, we demonstrated that miR-152-5p plays a vital role in ATG14-mediated autophagy, which further reduced the endogenous ROS accumulation and maintain cellar redox homeostasis (Fig. 7). All in all, we, at the first time, demonstrated that miR-152-5p/ATG14/ROS axis was involved in osteogenic differentiation which may contribute to osteoporosis. Therapeutic inhibition of miR-152-5p may be an efficient anabolic strategy for osteoporosis.

## Supplementary Information

**Additional file 1**. **Figure S1. **Establishment and validation of rat mandible osteoporosis model. (a) Three-dimensional (3D) reconstructive images and uCT images of sham and OVX rats' mandibular. The bone mineral density (BMD) of sham and OVX rats’ mandibular. trabecular bone thickness. (Tb.Th). The bone volume/the total volume (BV/TV) (n=3). (b) HE staining of sham and OVX rats' mandibular. (c) Characterization of MMSCs-M. MMSCs-M were positive for CD90, but negative for CD45, CD11b. (d) Oil Red O staining was applied to assess the ability of adipogenic differentiation of sham and OVX MMSCs-M. (e) Chondroblast staining of sham and OVX MMSCs-M. Scale bar=200um, data were shown as mean ±SD (p<0.05*, p<0.01**, p<0.0005***, p<0.0001****).

**Additional file 2**. **Figure S2.** (a). QRT-PCR was assessed the transfection efficiency of miR-152-5p mimics and inhibitor. (b). Western blotting assays were detected the expression of LC3B and p62 in control and NC and miR-152-5p mimics groups. (c). Immunofluorescence staining of LC3B in control, NC and miR-152-5p mimics group. (d–f). The ability of osteogenic differentiation was detected by Alizarin Red S staining, ALP staining and activity and western blotting in control, NC and miR-152-5p mimics groups. Data were shown as mean ±SD (p<0.05*, p<0.01**, p<0.0005***, p<0.0001****).

**Additional file 3**. **Figure S3.** (a). The transfection efficiency of siATG5. (b).The transfection efficiency of siATG14. (c). Western blotting of ALP, RUNX2 and OCN in groups of inhibitor NC + siNC, miR-152-5p inhibitor + siNC, inhibitor NC + siATG14, miR-152-5p inhibitor + siATG14. (d–e). QRT-PCR was used to examine the osteogenic differentiation and autophagy in the groups of NC+ATG14 NC, mimics+ATG14 NC and mimics+ATG14. Data were shown as mean ±SD (p<0.05*, p<0.01**, p<0.0005***, p<0.0001****).

## Data Availability

All data generated or analyzed during this study are included in this published article.

## Abbreviations

ATG5: Autophagy-related protein homolog 5
ATG14: Autophagy-related protein homolog 14
ALP: Alkaline phosphatase
BV/TV: Bone volume relative to total volume
BMD: Bone mineral density
BMMSCs: Bone mesenchymal stem cells
MMSCs-M: Mesenchymal stem cells from mandible
OP: Osteoporosis
OCN: Osteocalcin
OVX: Ovariectomy
PIK3C3: Phosphatidylinositol 3-kinase catalytic subunit type 3
ROS: Reactive oxygen species
Runx2: Runt-related transcription factor 2
Tb.Th.: Trabecular thickness
TEM: Transmission electron microscopy
