# Supplementary figures and images for: MiR-152-5p suppresses osteogenic differentiation of mandible mesenchymal stem cells by regulating ATG14-mediated autophagy
Source: Stem Cell Res Ther. 2022 Jul 26;13:359. doi: 10.1186/s13287-022-03018-4 (PMC9327198; doi:10.1186/s13287-022-03018-4)

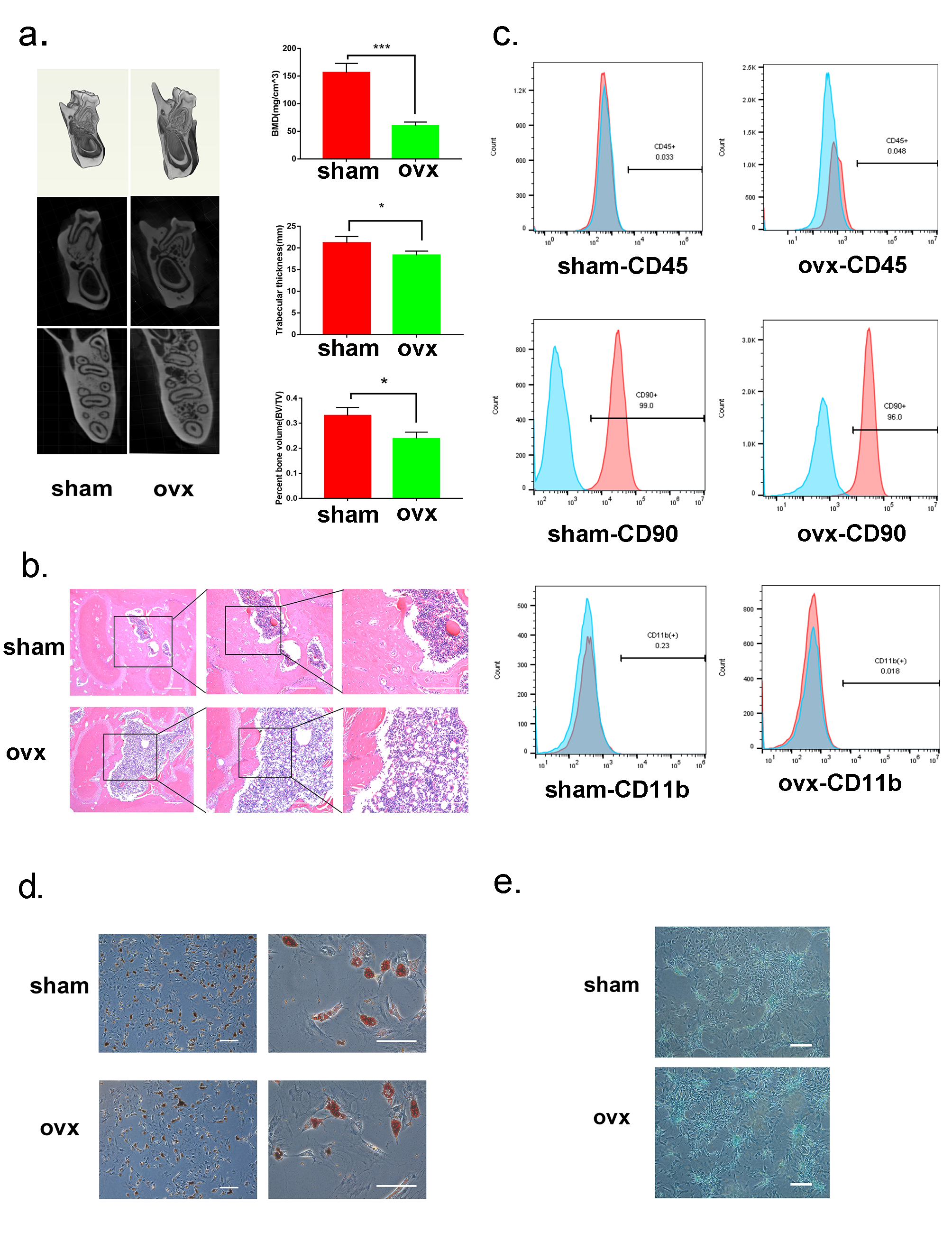

Supplement: Supplementary file 1 — Additional file 1. Figure S1. Establishment and validation of rat mandible osteoporosis model. (a) Three-dimensional (3D) reconstructive images and uCT images of sham and OVX rats' mandibular. The bone mineral density (BMD) of sham and OVX rats’ mandibular. trabecular bone thickness. (Tb.Th). The bone volume/the total volume (BV/TV) (n=3). (b) HE staining of sham and OVX rats' mandibular. (c) Characterization of MMSCs-M. MMSCs-M were positive for CD90, but negative for CD45, CD11b. (d) Oil Red O staining was applied to assess the ability of adipogenic differentiation of sham and OVX MMSCs-M. (e) Chondroblast staining of sham and OVX MMSCs-M. Scale bar=200um, data were shown as mean ±SD (p<0.05*, p<0.01**, p<0.0005***, p<0.0001****). [file 13287_2022_3018_MOESM1_ESM.tif]

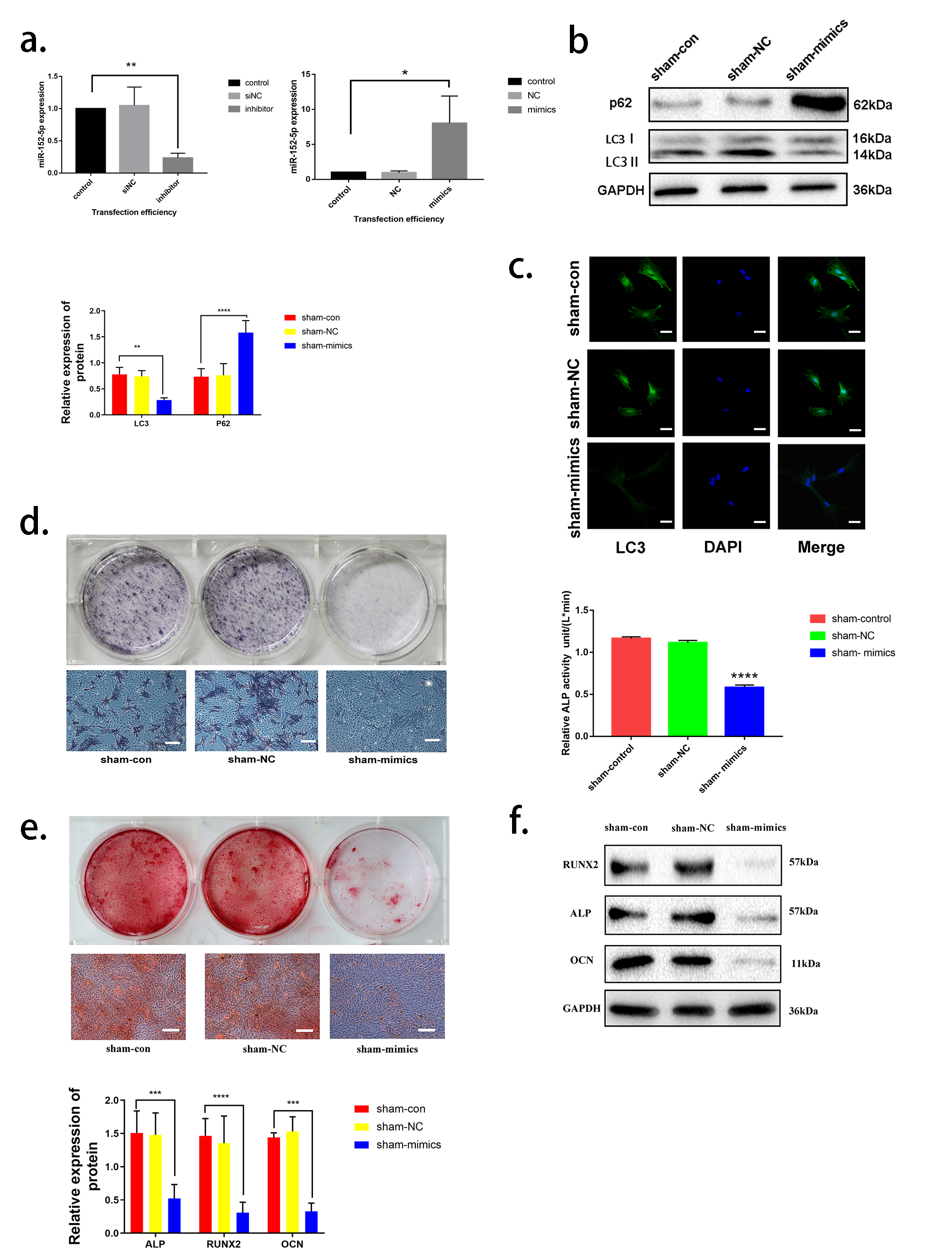

Supplement: Supplementary file 2 — Additional file 2. Figure S2. (a). QRT-PCR was assessed the transfection efficiency of miR-152-5p mimics and inhibitor. (b). Western blotting assays were detected the expression of LC3B and p62 in control and NC and miR-152-5p mimics groups. (c). Immunofluorescence staining of LC3B in control, NC and miR-152-5p mimics group. (d–f). The ability of osteogenic differentiation was detected by Alizarin Red S staining, ALP staining and activity and western blotting in control, NC and miR-152-5p mimics groups. Data were shown as mean ±SD (p<0.05*, p<0.01**, p<0.0005***, p<0.0001****). [file 13287_2022_3018_MOESM2_ESM.tif]

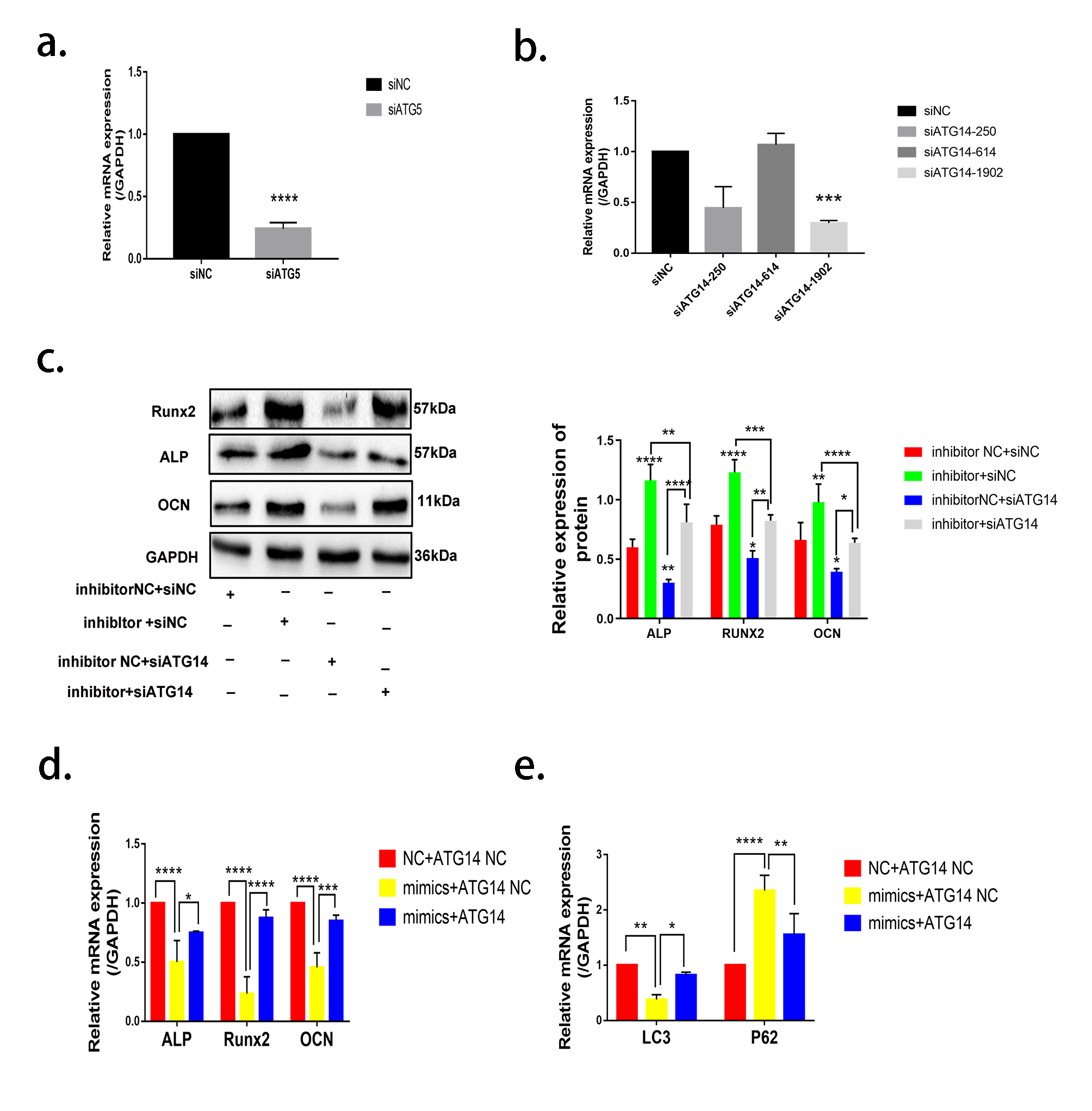

Supplement: Supplementary file 3 — Additional file 3. Figure S3. (a). The transfection efficiency of siATG5. (b).The transfection efficiency of siATG14. (c). Western blotting of ALP, RUNX2 and OCN in groups of inhibitor NC + siNC, miR-152-5p inhibitor + siNC, inhibitor NC + siATG14, miR-152-5p inhibitor + siATG14. (d–e). QRT-PCR was used to examine the osteogenic differentiation and autophagy in the groups of NC+ATG14 NC, mimics+ATG14 NC and mimics+ATG14. Data were shown as mean ±SD (p<0.05*, p<0.01**, p<0.0005***, p<0.0001****). [file 13287_2022_3018_MOESM3_ESM.tif]
